# Perceptions of adult Arkansans regarding trusted sources of information about the COVID-19 pandemic

**DOI:** 10.1186/s12889-021-12385-1

**Published:** 2021-12-20

**Authors:** Rachel S. Purvis, Don E. Willis, Ramey Moore, Cari Bogulski, Pearl A. McElfish

**Affiliations:** 1 College of Medicine, University of Arkansas for Medical Sciences Northwest, 1125 N. College Avenue, Fayetteville, AR 72703 USA; 2 Office of Community Health and Research, University of Arkansas for Medical Sciences Northwest, 1125 N. College Avenue, Fayetteville, AR 72703 USA

**Keywords:** COVID-19 information, Trusted sources of information, Health communications, Distrust

## Abstract

**Background:**

The United States leads the world in confirmed COVID-19 cases; Arkansas ranks fifth in average daily cases per 100,000. Historically, Americans relied on health communications from governmental sources and the news media. However, there has been a documented decline of trust in these sources. The present study seeks to understand trusted sources of information about COVID-19 to improve health messaging because research shows the level of trust is associated with adherence to recommendations.

**Methods:**

Data were collected using an online survey from participants (*N* = 1221) who were 18 years of age or older and residing, employed, or accessing health care in Arkansas. A qualitative descriptive design was used to summarize participants’ experiences and perceptions related to trusted sources of COVID-19 information.

**Results:**

Two primary themes related to participants’ perceptions of sources of information about COVID-19 are reported: 1) *trusted sources of information* and 2) *distrust or lack of trust in sources of information*. Several subthemes emerged within each primary theme. Results showed high trust in the academic medical center, federal and state public health agencies, and local health care providers. The study also documents diverging voices of distrust and uncertainty in making sense of contradictory information. Participants reported the main reason for their lack of trust was the rapidly changing information and the lack of consistency in information provided across sources.

**Conclusions:**

This finding provides insight into the importance of coordination between national, state, and local communications to bolster trust. Personal recommendations and testimonies from trusted health care providers and professionals could inform public health messaging interventions to increase vaccine uptake.

## Background

On January 22, 2020, the Centers for Disease Control and Prevention (CDC) reported the first laboratory-confirmed case of COVID-19 in the United States (US) [1]. In January 2021, the US had the highest number of confirmed COVID-19 cases in the world [2], and the state of Arkansas was ranked fifth in average daily cases per 100,000 [3]. Federal and state public health institutions and organizations are primary sources of information about mandates and recommended prevention efforts like masks, social distancing, and travel restrictions information during COVID-19 and prior health crises [4]. Historically, US residents have documented a high degree of trust in these federal and state sources [5, 6]. A recent survey of US adults (*N* = 718) reported that 69% of respondents wanted the scientific public health leadership (i.e., the CDC Director or National Institutes of Health [NIH] Director) to lead the US response to the COVID-19 outbreak [5]. While the literature reports that US adults have a moderate to high level of trust in federal and state public health and governmental sources of information [5, 6], there has been a documented decline in trust of these sources of information related to COVID-19 [7–10].

News media sources such as national and local television, newspapers, and radio have been important sources of information during previous outbreaks of infectious disease [7, 11, 12]. However, a 2020 study (*N* = 10,139) reported that less than half of US adults (49%) said the news media’s COVID-19 coverage has been largely accurate and that public perception of the news media has not changed significantly [13, 14]. Social media platforms like Facebook and Twitter have become another source for health information [7, 15]; however, reports have also shown a surge and spread of misinformation related to COVID-19 through social media [14, 16, 17].

Research has documented that the level of trust in sources of information was associated with adherence to recommendations [6, 18]. In order to successfully manage the COVID-19 pandemic, it is important to understand the most trusted sources of information about COVID-19 [19]. This study conducted a mixed methods examination of Arkansas residents’ perceptions regarding trusted sources of information about COVID-19 to improve health messaging and dissemination practices.

## Methods

### Study design and sample

Data were collected using an online survey that collected quantitative and qualitative data simultaneously. The volunteer research participant registry, ARresearch, was utilized for participant recruitment. The Translational Research Institute at the University of Arkansas for Medical Sciences (UAMS) developed and maintains ARresearch. By enrolling in the registry, participants agree to be contacted about opportunities to participate in research; ARresearch registry participants are representative of the ethnic and racial diversity of Arkansas [20]. A recruitment email was sent to potential participants inviting them to participate in a study about their perceptions and experiences related to the COVID-19 pandemic. Recruitment took place from July to August of 2020. A total of 4077 recruitment emails were sent to potential participants after invalid or undeliverable email addresses were removed. To be included in the study, participants verified that they were 18 years of age or older and residing, employed, or accessing health care in Arkansas before recording their consent. Informed consent was obtained from all participants. Screening questions asking for first and last name, date of birth, and email address were used to eliminate duplicates among potential participants. A $20 gift card was provided as remuneration to all participants who completed the online survey.

### Data capture

Participant consent and study responses were captured using REDCap (Research Electronic Data Capture). REDCap is a widely used web-based software created for research data capture and management [21, 22]. Participant demographics were captured using items from the Behavioral Risk Factor Surveillance System [23]. Participants were asked to indicate the level of trust they had in specific sources of information about COVID-19 using a four point Likert scale. To elicit more in-depth responses, three open-ended questions were also utilized that prompted participants to provide written descriptions of their perceptions and experiences related to trusted sources of information about COVID-19 in their own words. All study materials and procedures are approved by the UAMS Institutional Review Board (IRB#261226), and all methods were performed in accordance with the Declaration of Helsinki.

### Analytic strategy

Participant characteristics and perceived level of trust in sources of information about COVID-19 were tabulated and provided as descriptive summaries. A qualitative descriptive approach was utilized for data analysis of open-ended questions. This descriptive design synthesizes a summary of participants’ experiences and perceptions as well as the meanings that participants attribute to them [24–26]. All responses were reviewed by three qualitative researchers who then developed a codebook with emergent primary and subthemes. Data segments were coded, confirmation coding analysis was performed, and the codebook was revised three times with refined codes. The most illustrative quotes from the open-ended survey questions were sorted under each thematic domain. Participants provided over 3000 responses to these open-ended items, which required that only the most representative quotes be presented. Each analysis summary was critically reviewed by the research team to ensure that the data and illustrative excerpts were extracted to the appropriate thematic domain and to ensure analytic rigor and reliability. Any divergences in data interpretation were discussed by the research team and resolved via consensus. A descriptive qualitative design aims to allow participants to express themselves in their own voice and with their own words. Participants provided typed responses to open-ended survey questions, and the research team has retained the punctuation and capitalization to allow participants’ written inflection to be reflected as they presented it.

## Results

There were 1288 responses to the initial recruitment email invitation. Of those, 1221 participants met the inclusion criteria for participation in the study. Fifty-six participants were excluded because they did not meet the inclusion criteria, and eleven duplicate records were also excluded. Table 1 presents participants descriptive statistics for age, sex, race, income, and education. The majority of participants for the study were females (75.23%) and between the ages of 35 and 64 years old (58.59%). The majority of participants identified as White/Non-Hispanic (76.37%), and most participants (60.40%) were college graduates. The sample is diverse; however, it is over-representative of women and college graduates [27].

**Table 1:** Participant demographic characteristics

Variable	n (%) or Mean ± SD
Age (*n* = 1205)	48.1 ± 15.6
18-34	309 (25.6)
35-64	706 (58.6)
≥ 65	190 (15.8)
Sex (*n* = 1203)	
Women	905 (75.2)
Men	298 (24.8)
Race (*n* = 1202)	
Black	161 (13.4)
White	918 (76.4)
Other	43 (3.6)
Hispanic	80 (6.7)
Income (*n* = 974)	
< 25,000	205 (21.1)
25,000 to < 50,000	284 (29.2)
50,000 to < 75,000	221 (22.7)
> 75,000	264 (27.1)
Education (*n* = 1202)	
HS degree or less	145 (12.1)
Some college	331 (27.5)
College degree or more	726 (60.4)

There were two primary themes related to participants’ perceptions of and experiences with sources of information about COVID-19: 1) *trusted sources of information* and 2) *distrust or lack of trust in sources of information*. Several subthemes emerged within each primary theme and are presented with definitions in Table 2.

**Table 2:** Definitions and examples of primary themes and emergent subthemes

Theme	Subtheme
*Trusted sources of information*	*• Health care* *- Academic medicine and health sciences researchers* *- Local health care providers* Health care provider participants know personally Local health care providers working with COVID-19 patients *- Public health organizations and leaders* Federal State *• News media* *- Local* *- National*
*Distrust or lack of trust in sources of information*	*• Do not trust any sources about COVID-19* *• Lack of trust* *- In governmental sources of information* *- In media*

### Trusted sources of information

Table 3 presents the trusted sources of information reported by participants. UAMS, Arkansas’s only academic medical center, was documented as being the most trusted source of information, followed by federal health agencies and the Arkansas Department of Health (ADH). Social media and social media influencers had the lowest level of trust.

**Table 3:** Trust in sources of information about COVID-19

	Great extent	Somewhat	Very little	Not at all	Total
	%	%	%	%	%
^a^University of Ark for Medical Sciences	70.49	22.93	4.44	2.13	100.00
US Dept of Health, CDC, NIH, & other federal health agencies	65.75	23.39	7.77	3.09	100.00
Arkansas Dept of Health	61.77	28.14	6.99	3.10	100.00
My primary care provider	59.23	32.69	5.42	2.66	100.00
Other medical institution press releases	46.42	39.17	9.97	4.44	100.00
National television news	19.16	41.98	25.58	13.28	100.00
Local television news	17.20	50.72	23.21	8.87	100.00
Newspapers	15.76	47.39	23.21	13.64	100.00
Conversations with colleagues	12.29	50.46	27.06	10.18	100.00
Websites or online news pages	10.27	42.14	32.50	15.09	100.00
My pastor	10.19	32.29	26.97	30.56	100.00
Conversations with family and friends	7.90	44.19	36.73	11.18	100.00
Radio stations	6.54	35.01	37.35	21.10	100.00
Social media (e.g., Facebook, Twitter, YouTube, WhatsApp)	4.58	11.14	37.20	47.08	100.00
Celebrities and social media influencer	1.71	7.37	26.71	64.21	100.00

^a^Academic medical center and lead health research institute for the state of Arkansas

Note: US=United States, CDC=Centers for Disease Control and Prevention, NIH=National Institutes of Health

### Health care

Participants identified health care sources they trusted most for information about COVID-19. Participants’ qualitative responses identified: 1) academic medicine and health sciences researchers, 2) local health care providers, and 3) public health organizations and leaders as trusted sources of information about COVID-19. Participants’ qualitative responses provided nuance that helped explain why these particular sources were perceived as most trusted for COVID-19 information.

#### Academic medicine and health sciences researchers

Participants repeatedly described their trust in academic medicine and health sciences as trusted sources of information. Most participants (93.42%) reported the academic medical center as a source of information they trusted somewhat or to a great extent. Participants’ stated, “reliable medical professionals actively participating in clinical research and currently working in the field of epidemiology,” “medical professionals, virologists, epidemiologists, and infectious disease doctors,” and “physicians specializing in infectious diseases and epidemiology” were trusted sources of information. One participant succinctly explained, “if the information begins with virologists and people who have dedicated their lives/career to infectious disease prevention, they tend to always have my ear.” UAMS and Johns Hopkins were often identified by participants’ open-ended responses as medical institutions most trusted for COVID-19 information. Participants stated: “I trust UAMS and Johns Hopkins above all other sources” and that they read “daily updates from UAMS” about COVID-19. Another explained, “I trust Dr. Cam Patterson [Chancellor] at UAMS.” Other participants explained that they trust “Johns Hopkins for both accuracy and completeness (as much as possible) for US data,” “John Hopkins, Mayo Clinic, UAMS and other respected medical organizations,” “Johns Hopkins for tracking numbers,” and the “UAMS website” and “the ACHI [Arkansas Center for Health Improvement] website” for information about COVID-19.

#### Local health care providers

Participants frequently identified health care providers who they knew personally (i.e., family members, friends, and colleagues who are health care providers or the participants’ primary care physician or another health care provider who provide them with care). Participants reported trusting in information “somewhat” or “to a great extent” from their primary care provider (91.92%), and participants repeatedly identified “my doctor,” “my health care provider,” and “my primary care physician” as trusted sources for COVID-19 information. Participants expressed high confidence in their primary care provider as a trusted source of information, stating, “I would trust my doctor’s office to tell me about COVID-19 and any new information that came out about it” and “I trust my PCP, and would listen to her in almost any case I can think of.” Another participant explained, “I’ve spoken to my child’s pediatrician during a wellness visit and my gynecologist during my yearly appointment, and I trust both of them more than what I see on TV or read in the news.” Participants with chronic conditions identified specialists who provided them with care as a trusted source, with one participant saying, “Since I have MS I depend on my neurologist to provide me with relevant info.”

Participants identified local hospitals, local health care workers, and health care providers on the front lines treating COVID-19 patients as trusted sources of information. A participant expounded on their trust of local hospital, saying, “I trust local hospital and agencies more than federal because they take time to assess and properly filter and implement guidelines. Also, locally, they tend to be more preemptive in countermeasures than federal entities.” Additionally, participants stressed that “health care and medical professionals who are on the front lines and in the know,” “doctors and nurses on the front lines,” and “nurses who are working with COVID-19 patients” were trusted sources of information. A participant explained that these were trusted sources because “medical professionals who have been caring for COVID patients have a better understanding of what one needs to prevent the spread of the contagion.”

More than half of the participants reported conversations with colleagues (62.75%) and conversations with family and friends (52.09%) as trusted sources of information about COVID-19 either “somewhat” or “to a great extent.” The qualitative comments provided context demonstrating that participants were often referring to their family, friends, and colleagues who work in health care. One participant explained they, “talk to friends/family who are in health care.” Several participants specifically mentioned their family members who were health care providers as a trusted source: “My dad is a doc. I listen to him,” “My wife is a nurse so I trust her,” “my son and daughter-in-law, [who] are both doctors,” “Son (MD), daughter (MD),” “my daughter (RN),” and “My brother (MD),” were seen as trusted sources of information about COVID-19. A participant said, “Right now, only a trusted friend in the medical community,” and another participant stated, “Friends who are working with COVID who are RNs or MDs,” were their trusted sources of information.

#### Public health organizations and leaders

Participants expressed a high level of trust in federal and state public health organizations, and organizations such as the ADH, the CDC, Arkansas Governor Asa Hutchinson’s COVID-19 taskforce, and infectious disease expert Dr. Anthony Fauci as additional trusted sources.

Survey responses documented participants’ high level of trust (trusting “somewhat” or “to a great extent”) in sources from public health organizations and leaders at the federal (89.14%) level. In particular, the CDC was mentioned as a trusted source of information about COVID-19. Participants said, “I only trust scientifically valid outlets with reputations for helping like the WHO and CDC,” “For projections, and data extrapolation and imaging, I trust the CDC’s accuracy but not completeness,” and “[the] Center for Disease Control for recommendations for prevention and treatment.” Numerous participants identified specific federal public health officials as trusted sources of information. Participants said: “I trust Dr. Fauci,” “I listen to Dr. Deborah Birx and Dr Anthony Fauci,” and “Dr. Fauci has my complete confidence.”

Survey responses showed participants’ high level of trust (trusting “somewhat” or “to a great extent”) in sources from public health organizations and leaders at the state (89.91%) level. Participants expressed high levels of trust in the ADH and its leadership. A participant said, “I trust Drs. Smith and Romero at the State Health Dept.,” and another stated, “The Arkansas Dept. of Health is about the only one.” The ADH COVID-19 dashboard was another source that many participants identified: “I also check Arkansas’ health department website;” “Arkansascovid.com has been invaluable for a reliable, independent perspective on ADH’s public data;” and “arkansascovid.com is trustworthy and responsible, with no political agenda. I generally trust the Department of Health.” Arkansas Governor’s COVID-19 taskforce and its daily briefings were also highlighted by participants as a trusted source for information. Many participants said, “I watch the governor’s press conference every day,” or “I listen to the governor’s report daily.” A few expounded on why they trusted this particular source. One participant said, “I trust our governor, Asa Hutchinson and Nate Smith [ADH Secretary] to let us know what’s happening in our state.” Another participant stated, “I watch Governor Hutchinson’s new conferences almost daily and trust what he is telling us. I believe he is trying to provide a safe environment for our state.” Another participant explained, “I listen to the governor’s daily briefings because this gives me information about things that are being done that will affect my life in practical ways, but I am always thinking about motives and pragmatics.”

### News media

Participants’ survey responses demonstrated a moderate level of trust (i.e., responding “somewhat” or “to a great extent”) in local television news (67.92%), newspapers (63.15%), national television news (61.14%), websites or online news pages (52.41%), and radio stations (41.55%). Participants reported a lower level of trust in social media (e.g., Facebook, Twitter, YouTube, and WhatsApp) (15.72%). A participant explained, “I feel like the local news is about the only source we can hear from/trust.” Other participants identified specific local television news stations including, “THV11 news station,” “local THV news,” and “Channel 7 news.” Specific local and national newspapers were also identified, with one participant stating, “I read the [Arkansas] *Democrat Gazette* for information regarding the rest of USA and the world” and another saying they most trusted “reputable news sources such as *New York Times* or *Washington Post*.” Participants also identified various national news media sources they trusted for COVID-19 information. One person explained, “I mostly listen to NPR and local new channels. Lately it’s mostly been NPR and trusted new sites from twitter.” Another person said that they most trusted information from “Medical updates (I am an FNP), and world news report at 5:30 week days, Fox News Updates and specials.” Participants also said, “I read CNN for more information that I trust,” and “I trust some news reports from online sources,” and that “official news sources [like] (CNN, FOX, NBC, CBS, etc.)” were trustworthy.

### Distrust or lack of trust in sources of information

While many participants expressed a high level of trust in health care providers and a moderate level of trust in news media, there were also participants who described a distrust or lack of trust in some or all of the aforementioned information sources.

#### Do not trust any sources about COVID-19

Some participants reported that they did not trust any sources or were unsure which sources could be trusted for information about COVID-19. Participants said, “I’m not sure who to trust for Covid-19 information,” “I don’t really trust anybody or any source at this time,” and “Sadly I am not sure who to trust at this point.” Other participants expressed skepticism about COVID-19 and its seriousness. For example, one participant explained, “None...pretty sure this virus is made up and it’s being used to freak people out.”

Participants explained that one of the reasons they distrusted sources about COVID-19 was because information concerning COVID-19 was rapidly changing, contradictory, and confusing. Participants explained, “We have been told so many things, often at great variance, and I have no way of knowing what to believe,” and another stated, “Honestly I am not too sure about any of the information out there. All the sources are saying different things and I don’t know who to trust.” The rapidly changing nature of a pandemic was mentioned by a majority of participants as a main source of their distrust. Participants explained, “I’m not sure I trust anyone right now because all the agencies seem to be changing opinions on COVID daily;” “There are no trust worthy sources as the information and knowledge base is constantly changing;” and “Really hard to ‘trust’ anyone -- everything changes from day to day.”

Participants stated that COVID-19 information was often confusing and contradictory, stating, “All messaging are confusing and there is no set path to get virus under control,” and “We have received conflicting messages from the beginning.” The lack of agreement among sources was described as creating distrust. Participants said, “There are zero sources on COVID-19 that are trustworthy due to the varying differences of opinion, supposed truths that later become falsehoods and no agencies in total agreement with one another,” and “I don’t know anymore. There is so much info of back in forth. Do or don’t, yes or no. I feel I am high risk, and the uncertainty is scaring me.” Other participants explained, “Honestly I am not too sure about any of the information out there. All the sources are saying different things and I don’t know who to trust,” and “There is a lot of disinformation out there, but, even from trusted sources, constant changes in procedures and precautions do not help in maintaining trust.” This participant summarized the source of their distrust: “I do not trust anyone or source because the sources that we’ve all been seeing have changed the information too much. I believe that after a couple years of study we will have a greater understanding of what really will prevent spreading or getting this virus.”

Participants noted difficulty in determining the trustworthiness of a source due to misinformation and a lack of clear, coherent health messaging. A participant explained, “This is a very hard question to answer. We have had so much conflicting and every changing opinions from every source that really all we can do is put it all together and decide for yourself what is real and what is not real.” Other participants explained that there is “So much misinformation and rumors that it is hard to separate the truth from falsehood,” and “I am not convinced that there are any trusted sources for COVID-19. Opinions and stories are all over the place. You can find what you want to believe, without regard to truth and accuracy.”

#### Lack of trust in governmental sources of information

While many participants identified public health sources like the CDC, ADH, and NIH as trusted sources, there were divergent voices. Some participants described a lack of trust in state and federal governmental sources of information. One participant succinctly stated, “I especially do not trust the CDC, the NIH, and other government connected health sources.” Several participants mentioned the politicizing of COVID-19 and COVID-19 messaging had greatly weakened their trust for official sources. Participants explained, “It’s hard to trust anything because of the political skew and constant changing of information,” and “I have lost faith in full disclosure and keeping it real from our governments.” Many participants expressed a complete lack of trust in official governmental sources: “I definitely do NOT trust politicians in DC or our governor. They do not have our best interests in mind when making politically charged statements and decisions regarding our health,” and “I have no trust in our federal government officials to provide the truth to us unfortunately.” One participant explained, “Trusted sources are primarily those with relevant knowledge that are both inquisitive and informative regarding this virus. People with ‘skin in the game’ even with information cannot be trusted as having public interest at heart.”

Some participants described their lack of trust in CDC messaging about COVID-19. Participants said, “I would like to say the CDC, however, contradictories have made me second guess them.” Others expressed that the lack of trust in the CDC was a result of political pressure. Participants explained, “I am afraid to trust the CDC because of the pressure I worry they are under because of our president,” and “I use to trust the CDC but there has been political interference.” Another participant said, “I generally trust the CDC, but the CDC has been compromised by the White House in a number of unfortunate ways from underfunding to censoring their reports and bypassing their data.” Participants stated they use to trust “Dr. Fauci [and] the CDC before Trump hushed them,” and “Not sure anymore since Dr. Fauci and the CDC has issued so many statements that seem to contradict either their own previous statements or guidelines issued before.”

Some participants reported a lack of trust in official state government sources such as the ADH and the governor. One participant said, “I generally trust the Department of Health, but Arkansas Department of Health has been too eager to spin their data politically to create a rosier picture for the governor’s daily briefings.” Other participants said, “Arkansas Department of Health seems to have conflicting information in the press releases they do with the governor,” and “I do not trust the governor, especially when he is involved with Arkansas Department of Health.” One participant explained their lack of trust in official state sources: “There is no guidance from state government only a recitation of statistics.” Others expressed concern about the accuracy of data reported by official state sources. One participant said, “The governor gives us information that has the numbers skewed. I am educated enough I know how to read the graphs and understand per capita. But the governor is pandering to those who take things for face value. We need more numbers out there for people to know how bad this is.” Another explained, “The data being released by our governor doesn’t seem to paint the full picture of the virus spread, so I try to get my news from less conservative sources that are more grounded in science and facts.”

#### Lack of trust in media

Numerous participants identified and explained their lack of trust in media as a source of information about COVID-19. Participants said they “Don’t trust media at all,” they “Do not trust any American medical, or media source,” and “I do not trust the mainstream media.” Another participant explained that, “Newscasters are no longer objective, and FOX news is completely unreliable, so it’s hard to know who to trust. It seems people have no honor anymore and would sell their souls for a buck, regardless of the consequences to the country, their family, themselves, or the future. This causes general distrust of many.” However, one participant stated, “Right now I do not trust ANY main stream news source. I do think Fox news provides the most accurate information because they do not have a political agenda to get Pres. Trump out of office.” One participant said, “Good question! Definitely NOT the media outlets. However, it is an ever-changing informational model that is likely to be revised. Perhaps if it wasn’t so politicized, I would be more likely to take stock on what I’m learning from medical sources from this virus.” Another participant said, “I’m not sure who to trust. I feel the media is very involved in trying to scare everyone. I don’t trust the statistics being published as I have heard too much about false positives.”

## Discussion

This study documents participants’ trust and lack of trust in COVID-19 information using qualitative and quantitative methodology. Most participants articulated trust in health care sources of information related to COVID-19, with particular trust in the academic medical center, federal and state public health agencies, and local health care providers they know. These findings are consistent with prior literature that has documented high levels of trust in federal public health organizations like the CDC [5, 6, 8] and health care providers [28]. These finding are consistent with prior research that found doctors were the most trusted source of information about COVID-19 and may play a role in addressing COVID-19 conspiracy theories [28]. Participants of this study expressed their trust in COVID-19 information from health care providers that they received care from as well as providers that they had personal relationships with such as family members, trusted friends, and colleagues. This is the first qualitative study that adds further nuance to these findings by documenting that participants report they trusted doctors and other medical professionals that they knew personally. Health care provider recommendations are one of the most effective interventions for increasing vaccine uptake [29]. Findings may inform public health messaging campaigns that incorporate personal testimonies related to experiences of the COVID-19 pandemic from trusted health care providers and medical professionals to increase vaccine uptake in other states, like Arkansas, which report large numbers of COVID infections and low vaccination rates.

Approximately half of the participants reported trust in news media, and most participants reported a low level of trust in social media as a source of information about COVID-19. These findings are consistent with previous studies that have documented a deterioration of the trust in news media [13] and findings that document news media is often seen as a less trustworthy source of COVID-19 information compared to other sources [6–8].

Despite participants reporting high levels of trust in health care providers and a moderate level of trust in the news media, there were divergent views. Some participants expressed a distrust or lack of trust in any source of information regarding COVID-19. Participants identified specific previously trusted public health sources (e.g., CDC, NIH, and ADH) that they now considered untrustworthy. These findings are consistent with previous findings documenting a steady decrease in American trust in COVID-19 information from federal and state governments since March 2020 [7–10]. The primary reason participants gave for their lack of trust was the rapidly changing information and the lack of consistency in the information provided across sources. This is the first time this finding has been documented in participants’ own words. This is a particularly significant finding and provides insight into the importance of coordination between national, state, and local organizations to bolster trust.

## Limitations

This study is not without limitations. Participants were recruited from a research registry in Arkansas and may not be representative of the general US population. This weakness is mitigated somewhat because participants in the registry are representative of the racial and ethnic diversity of Arkansas; however, the sample is over-representative of women and college graduates in the state. While the open-ended responses allowed each participant to type out their responses in their own words with greater anonymity, researchers did not have the opportunity to probe for clarification of participants’ written responses. Future research should examine if those with lower educational attainment report high levels of trust in health care providers and professionals as sources of COVID-19 information.

## Conclusion and implications

This study makes a significant contribution to the literature on trusted sources of information for COVID-19. This study documents high trust in the academic medical center, federal and state public health agencies, and local health care providers. The study also documents diverging voices of distrust and uncertainty in making sense of contradictory information. The findings suggest that trust could be enhanced by ensuring consistent messaging among health care information sources. These findings contribute important nuance about participants’ trusted sources of COVID-19 information that can better inform future health messaging campaigns and policies that incorporate personal recommendations from trusted health care providers and professionals into interventions to increase vaccine uptake.

## Data Availability

The data that support the findings of this study are not made publicly available due to regulatory restrictions but are available from the corresponding author upon reasonable request.

## Abbreviations

ACHI: Arkansas Center for Health Improvement
CDC: Centers for Disease Control
US: United States
NIH: National Institutes of Health
UAMS: University of Arkansas for Medical Sciences
REDCAP: Research Electronic Data Capture
ADH: Arkansas Department of Health
